# Intranasal Administration of Mesenchymal Stem Cells Ameliorates the Abnormal Dopamine Transmission System and Inflammatory Reaction in the R6/2 Mouse Model of Huntington Disease

**DOI:** 10.3390/cells8060595

**Published:** 2019-06-15

**Authors:** Libo Yu-Taeger, Janice Stricker-Shaver, Katrin Arnold, Patrycja Bambynek-Dziuk, Arianna Novati, Elisabeth Singer, Ali Lourhmati, Claire Fabian, Janine Magg, Olaf Riess, Matthias Schwab, Alexandra Stolzing, Lusine Danielyan, Hoa Huu Phuc Nguyen

**Affiliations:** 1 Institute of Medical Genetics and Applied Genomics, University of Tuebingen, D-72076 Tuebingen, Germany; Libo.Yu-Taeger@med.uni-tuebingen.de (L.Y.-T.); janice.strickershaver@gmail.com (J.S.-S.); Patrycja.Bambynek-Dziuk@med.uni-tuebingen.de (P.B.-D.); Arianna.Novati@med.uni-tuebingen.de (A.N.); Elisabeth.Singer@med.uni-tuebingen.de (E.S.); Janine.Magg@med.uni-tuebingen.de (J.M.); Olaf.Riess@med.uni-tuebingen.de (O.R.); 2 Centre for Rare Diseases (ZSE), University of Tuebingen, D-72076 Tuebingen, Germany; 3 Interdisciplinary Centre for Bioinformatics (IZBI), University of Leipzig, D-04107 Leipzig, Germany; katrin.arnold@izi.fraunhofer.de (K.A.); claire.fabian@izi.fraunhofer.de (C.F.); A.Stolzing@lboro.ac.uk (A.S.); 4 Fraunhofer Institute for Cell Therapy and Immunology (IZI), D-04103 Leipzig, Germany; 5 Department of Clinical Pharmacology, University Hospital of Tuebingen, D-72076 Tuebingen, Germany; alilourhmati@yahoo.de (A.L.); Matthias.Schwab@ikp-stuttgart.de (M.S.); Lusine.Danielyan@med.uni-tuebingen.de (L.D.); 6 Dr. Margarete Fischer-Bosch Institute of Clinical Pharmacology, D-70376 Stuttgart, Germany; 7 Departments of Biochemistry and Clinical Pharmacology, Yerevan State Medical University, 0025 Yerevan, Armenia; 8 Laboratory of Neuroscience, Yerevan State Medical University, 0025 Yerevan, Armenia; 9 Centre for Biological Engineering, School of Mechanical, Electrical and Manufacturing Engineering, Loughborough University, Loughborough LE11 3TU, UK; 10 Department of Human Genetics, Ruhr University of Bochum, D-44801 Bochum, Germany; 11 Departments of Medical Chemistry and Biochemistry, Yerevan State Medical University, 0025 Yerevan, Armenia

**Keywords:** Huntington disease, cell therapy, mesenchymal stem cells, intranasal, R6/2 mice, dopamine transmission, microglia, neuroinflammation

## Abstract

Intrastriatal administration of mesenchymal stem cells (MSCs) has shown beneficial effects in rodent models of Huntington disease (HD). However, the invasive nature of surgical procedure and its potential to trigger the host immune response may limit its clinical use. Hence, we sought to evaluate the non-invasive intranasal administration (INA) of MSC delivery as an effective alternative route in HD. GFP-expressing MSCs derived from bone marrow were intranasally administered to 4-week-old R6/2 HD transgenic mice. MSCs were detected in the olfactory bulb, midbrain and striatum five days post-delivery. Compared to phosphate-buffered saline (PBS)-treated littermates, MSC-treated R6/2 mice showed an increased survival rate and attenuated circadian activity disruption assessed by locomotor activity. MSCs increased the protein expression of DARPP-32 and tyrosine hydroxylase (TH) and downregulated gene expression of inflammatory modulators in the brain 7.5 weeks after INA. While vehicle treated R6/2 mice displayed decreased Iba1 expression and altered microglial morphology in comparison to the wild type littermates, MSCs restored both, Iba1 level and the thickness of microglial processes in the striatum of R6/2 mice. Our results demonstrate significantly ameliorated phenotypes of R6/2 mice after MSCs administration via INA, suggesting this method as an effective delivering route of cells to the brain for HD therapy.

## 1. Introduction

Huntington disease (HD) is an autosomal dominant neurodegenerative disorder that affects 4–10 individuals per 100,000 [1,2,3,4]. It is an adult-onset, chronically progressing disease manifested by motor dysfunction, cognitive decline, and psychiatric symptoms together with weight loss and sleep disturbance (reviewed in [5,6]). HD is caused by an expansion of the CAG (coding for glutamine) repeat region in exon 1 of the huntingtin (*HTT*) gene that encodes the huntingtin protein (HTT) [7]. In mutant HTT (mHTT), the polyglutamine tract contains more than 38 glutamines and the length of the tract correlates inversely with the age of disease onset, with longer tracts resulting in earlier onset [3,4]. The neuropathological hallmarks of HD feature a substantial accumulation of protein aggregates containing truncated N-terminal mHTT fragments in the cortex and striatum [8], and striatal atrophy that progressively extends to cerebral cortex and other brain regions [9,10].

At present, there is no effective treatment for disease prevention or slowing down disease progression [11,12]. Existing medications are limited and only alleviate the HD symptoms so as to improve the quality of life of the patients [3,12,13], but do not extend the life span of the patients. Recent therapeutic development for neurologic disorders explored the potentials of multipotent mesenchymal stem cells (MSCs) that possess regenerative properties and their preferential tropism to migrate to damaged brain regions in the degenerating central nervous system (CNS) [14,15]. In vivo testing reported that the therapeutic effects of MSCs are mainly attributed to their neuroprotective/immunomodulatory capacity and enhanced availability of bioactive factors including trophic and growth factors that could induce tissue repair and angiogenesis [16,17]. The therapeutic effects of MSCs were explored by intracerebral transplantation in animal models of HD [14,18,19,20,21], Parkinson’s disease (PD) [22,23,24,25,26,27] and Alzheimer’s disease (AD) [28,29,30,31,32,33,34,35,36], all of which ameliorated phenotypic impairments in MSC-treated animal models. It is, however, considered to have a limited translational potential [16]. While intracranial delivery enhances the number of cells reaching the targeted brain region when compared to systemic administration, the invasive nature of the delivery method poses high risk to the subject and restricts repeated cell administrations within a short period of time [37,38]. Later studies have hence utilized the innovative, non-invasive intranasal administration route for brain targeting [39,40]. We have previously shown that after MSCs crossed the cribriform plate, they either migrated into the olfactory bulb and subsequently to the other brain regions, or entered the cerebrospinal fluid (CSF) with movement along the surface of the cortex and then into the brain parenchyma [41], which has been recently confirmed [42]. Later we demonstrated the efficacy of intranasally administered MSCs in the 6-hydroxydopamine (6-OHDA) rat model of PD [43]. Likewise, beneficial effects of intranasally delivered MSCs were also reported in a rotenone-induced PD mouse model [44] and a spinal cord-lesioned rat model [45]. Based on the promising in vivo data and our technical expertise on intranasal MSC-treatment in neurological disease models, in this study we evaluated the therapeutic effects of MSCs administered via the intranasal route in HD using the R6/2 mouse model.

The R6/2 mouse model carries an N-terminal exon 1 fragment of the disease-causing human *HTT* gene that contains approximately 145 CAG repeats (length of polyglutamine expansion varies due to germ line instability) [46,47]. As a result, they display physiological and behavioral phenotypes that recapitulate symptoms of HD patients [48,49], including progressive weight loss, shortened life span [46,50,51], progressive motor dysfunction [50,52], cognitive decline [53,54] and neuropsychiatric-like disturbances [55,56] such as disrupted circadian rhythm [57]. Brain volume reduction and neuronal intranuclear inclusions are also consistently observed in R6/2 mice, resembling the neuropathological features of human HD [46,51,52]. Furthermore, R6/2 mice have been reported to have a wide range of gene dysregulation in various brain areas. This includes the expression of multiple inflammation- and stress-related genes as well as genes related to neurodegeneration [58]. As in other neurodegenerative diseases, neuroinflammation was detected in HD patients as well as in HD animal models like the R6/2 mice [59,60,61,62,63,64,65], in which pro-inflammatory cytokines such as interleukin 6 (IL-6) and tumor necrosis factor alpha (TNFα) were significantly elevated. It is well known that MSCs exert immunomodulatory effects by affecting immune T- and B-cell responses, including suppression of T- and B-cell proliferation and the regulatory response of the T-cell, as well as activation of dendritic and natural killer cells [66,67,68,69,70]. Moreover, MSCs secrete various cytokines, trophic and growth factors that support neuronal survival and regeneration [71,72]. Cell migration deficits including impaired function of microglia and the decreased expression of microglia marker Ionized calcium-binding adapter molecule 1 (Iba1) have been observed in HD transgenic mice [73,74]. Besides, the dopaminergic neurotransmission system is also severely impaired [75,76], as shown by the decreased mRNA expressions of both D1 and D2 dopamine receptors and their electrophysiological responses to receptor activation [77].

In this study, MSCs isolated from the bone marrow of young eGFP mice were transplanted into the transgenic HD mouse model R6/2 via the intranasal delivery route at the early disease stage. MSCs were found to have a dynamic and widespread distribution in several major brain regions. Physiological and behavioral parameters were monitored in MSC-treated R6/2 mice longitudinally post-transplantation and were compared to the control groups (PBS-treated wild type (WT) and PBS-treated R6/2 mice). We found that intranasal MSC treatment extended the life span and alleviated the circadian activity disruption of the R6/2 mice. Expression analyses revealed that these functional improvements were attributed to ameliorated neuroinflammatory activation and improved dopaminergic signaling. Moreover, MSCs could restore the expression of Iba1 as a marker of microglia and the morphology of striatum-resident microglia in R6/2 mice. Altogether, our study provides evidence that intranasal administration of MSCs is an efficacious delivery route for HD treatment and has a high translational potential to the clinics for HD as well as other neurodegeneration-targeting therapies.

## 2. Materials and Methods

### 2.1. Isolation, Cultivation and Characterization of MSC in Vitro

Transgenic mice expressing eGFP (8–12 weeks old, male, C57Bl/6-Tg(UBC-GFP)30Scha/J (eGFP mice) were obtained from Jackson Laboratories (Bar Harbor, ME). Bone marrow was harvested from tibia and femur as described previously [78]. MSCs were cultivated in minimum essential medium (MEM) α, GlutaMAX™ (Gibco, 32561029) with 15% fetal calf serum (FCS) (Gibco, 10270106) and 1% penicillin/streptomycin (Gibco, 15070-063) supplemented with 20 ng/mL FGFb (Peprotech, 450-33). MSCs were harvested at passage 2 and frozen in 10% DMSO/90% cultivation medium until transplantation. All MSCs used for transplantations were at passage three. Cells were harvested at passage four and fixed with 2% (*v/v*) buffered paraformaldehyde (Pierce, 16% Formaldehyde, Methanol-free) for 15 min at room temperature. Mouse Mesenchymal Stem Cell Marker Antibody Panel (R&D Systems, SC018) was used according to the manufacturer’s protocol. The panel consisted of the following antibodies: Anti-CD11b, anti-CD45, anti-Sca-1, anti-CD 106, anti-CD105, anti-CD73, anti-CD29, and anti-CD44, rat IgG2A (MAB006, Life Technologies) and rat IgG2B (MAB0061, Life Technologies). MSC were blocked in 5% BSA for 30 min at room temperature and then incubated with primary antibodies for 30 min at room temperature. MSC were washed two times with PBS and stained with secondary antibodies (1:200 dilution: Donkey anti-rat Cy3 (712-165-153, Dianova) or sheep anti-rat-NL557 (NL013, R&D)). After 30 min incubation at room temperature, cells were washed two times and fluorescence was measured using BD-Influx. Gates were set according to appropriate isotype controls. Dot blot graphs were created using BD FACS™ Software.

### 2.2. HD Animals

For the animal experiments, female mice expressing exon 1 of mutant human *HTT* gene with approximately 145 CAG repeats were housed with littermates of mixed genotype in groups of four with 12 h light/dark cycle and free access to food and water. All experiments were approved by the local ethics committee at the Regierungspraesidium Tuebingen (License Number:PH8/13), and carried out in accordance with the German Animal Welfare Act and the guidelines of the Federation of European Laboratory Animal Science Associations based on European Union legislation (Directive 2010/63/EU).

Breeding was performed by crossing wild-type B6CBAF1/J males with ovary-transplanted R6/2 females (B6CBA-TgN(HDexon1)62Gbp/J) supplied by The Jackson Laboratory (Charles River Laboratory). Genotyped female R6/2 and wild-type (WT) littermates from each cohort were assigned to different treatment groups according to their body weight and rotarod test performance to counterbalance the potential litter effects. Animals were divided into three treatment groups and recruited to all behavioral experiments: (1) R6/2 mice treated with MSCs resuspended in phosphate-buffered saline (PBS) (R6/2-MSC); (2) R6/2 mice treated with PBS (R6/2-PBS) and; (3) WT mice treated with PBS (WT-PBS) (*n* = 16 per group). Animals were sacrificed at 7.5 weeks after intranasal MCS vs. PBS treatment. For the analysis of cell migration in the brain animals were sacrificed five days post-delivery of MSCs (*n* = 3).

### 2.3. Intranasal Cell Transplantation

Mice at four weeks of age were administered with MSCs of passage three as previously described [41]. The mice were held with a hand grip that allowed the animals to recline on their backs while immobilizing the skull, and the nose drop containing the substance/cell suspension was carefully placed on one nostril allowing it to be snorted naturally, and then the other nostril. One hundred units of hyaluronidase (Sigma-Aldrich Chemie GmbH, H3506) dissolved in 24 µL sterile PBS was administered to the mouse nostrils (6 µL/nostril, repeat once after 2 min) 30 min prior to the administration of MSCs or PBS. One million of vital MSCs were freshly prepared from frozen stocks and resuspended in 24 µL of sterile PBS and applied to each mouse in the R6/2-MSC group using the same method as described for hyaluronidase, while R6/2-PBS and WT-PBS groups received the same amount of PBS only. Since the amount of living cells after the thawing procedure was highly variable for eGFP-MSC (50–75% survival), we thawed an excess of MSC, i.e., up to 2.5 × 10^6^ cells. This ensured that the total number of cells applied contained 1 × 10^6^ living cells, which was determined by the trypan blue staining immediately before cell administration. After three days, the administration was repeated so that each mouse in the R6/2-MSC group received two million of cells in total, whereas mice in the control groups received 24 µL of vehicle buffer (PBS) for the second time.

### 2.4. Rotarod Test

R6/2-MSC and controls R6/2-PBS and WT-PBS were tested at 6, 8 and 10 weeks of age (2, 4 and 6 weeks after transplantation) on a rotarod apparatus (AccuScan Instruments). Mice were tested over 3 consecutive days [79]. On each day, the animals received a training trial of 5 min at 4 rpm on the rotarod. One hour later, the animals were tested for 3 consecutive accelerating trials of 5 min with the speed changing from 4 to 40 rpm over 360 s and a minimum of 30 min inter-trial interval. The latency to fall from the rotating rod was recorded. Mice remaining on the rod for more than 360 s were removed and their time scored as 360 s.

### 2.5. Locomotor Activities and Food Intake

Locomotor activities and feeding behavior were monitored by the LabMaster system which provided a home cage-like environment embedded in an infrared light frame (TSE system GmbH). Animals were monitored for 22 h at 5 and 11 weeks of age (*n* = 15), and the data were collected automatically with 1 min intervals. As the animals were habituating to the new environment during the first two hours, these data were excluded from the analysis. Ambulatory activity was defined by the number of beam breaks along the x and y axes (horizontal activity), while beam breaks on z level were calculated as rearing (vertical activity). Fine movement was defined by repetitive beam breaks. Data were analyzed either by summating all activities in both phases as total activity or in the light phase and dark phase individually. Food intake was calculated as the food consumption over 22 h.

### 2.6. Quantitative PCR

RNA was extracted from mice tissues using peqGOLDTrifast^TM^ reagent according to the manufacturer′s instructions (PeqLab, 30-2040) and treated with DNase I (Life Technologies, EN0521). cDNA synthesis was performed using Superscript^TM^ III Reverse Transcriptase (Life Technologies, 18080085) and Oligo(dT)_18_-Primer (Thermo Scientific, SO132) at 50 °C for 1 h. cDNA (1:10 dilution) was used as PCR template with technical triplicate for every sample. Quantitative PCR was performed using the DNA engine CFX Connect™ Real-Time PCR Detection System (Biorad) according to published protocols [80].

### 2.7. Immunohistochemical Staining and Immunofluorescence Staining

Immunohistochemical analysis was performed on 11.5-week-old mice (7.5 weeks after MSCs administration). Mice were perfused transcardially with 4% paraformaldehyde in PBS (pH 7.4) and post fixed in the same fixatives overnight at 4 °C. Brains were serially cut into 25 µm-thick coronal sections, in which every 6th brain section was taken and pre-mounted on slices. All staining procedures were performed at room temperature. For the immunohistochemical staining, brain sections were incubated in 0.5% NaBH_4_ for 30 min for blocking. After washing, the sections were permeabilized in 0.3% Triton X-100 in TBS buffer (25 mM Tris-HCl, 137 mM NaCl, 2.7 mM KCl). For staining mHTT aggregates, primary antibody EM48 (Millipore, MAB5374) incubation was carried out overnight at a concentration of 1:1000, followed by incubation with biotinylated anti-mouse antibody (1:500, Vector Laboratories, BA9200) for 2 h. Avidin-biotin complexes (1:200, Vector Laboratories, PK6100) with a single round of biotinylated tyramine amplification were used to enhance the signal intensity. For color development, sections were exposed to nickel-DAB-H_2_O_2_ (0.6%/nickel sulfate, 0.01% 3,3-diaminobenzidine (DAB), and 0.001% hydrogen peroxidase) until they reached an optimal staining intensity. For the immunofluorescence staining, brain sections were blocked with 5% normal goat serum (Vector Laboratories, S-1000), and incubated in one of the following primary antibodies: Anti-dopamine and cyclic AMP-regulated phosphoprotein (DARPP-32) at a concentration of 1:1000 (Epitomics, 1710-1), anti-eGFP at a concentration of 1:250 (NovusBio, NB600-308), anti-Iba1 at a concentration of 1:2000 (Wako, 019-1974), and anti-neuron-specific nuclear protein (NeuN) at a concentration of 1:200 (Merck Millipore, MAB377B) overnight. The secondary antibody anti-Rabbit Alexa 594 was used at 1:500 (Dianova, 711-585-152).

### 2.8. Quantification of Striatal Area

To compare the striatal volume, brain sections of WT-PBS, R6/2-PBS and R6/2-MSC mice were stained using anti-DARPP-32 to visualize striata (*n* = 4). Six sections containing the striatum starting from approximately Bregma 0.98 (2 sections are ~150 µm apart) were chosen for quantification. Images were analyzed by ImageJ (National Institutes of Health, USA) and the striatal area of each brain section was defined by the DARPP-32-positive area. The striatal area of each animal was calculated as the average of the striatal area of the 6 brain sections analyzed.

### 2.9. Western Blotting Analysis

Mice striatal tissues were homogenized in ice-cold 10 volumes *w/v* modified RIPA buffer (150 mM sodium chloride, 1.0% NP-40, 0.5% sodium deoxycholate, 0.1% SDS, 50 mM Tris, 5 mM EDTA pH 8.0) with Complete Proteinase Inhibitor Cocktail tablets (Sigma-Aldrich, 1873580) with a mechanical homogenizer. After a further 5-min sonication step with a bath sonicator for shearing genomic DNA, the lysates were centrifuged at 16,200× *g* at 4 °C for 20 min to isolate the soluble protein. Protein samples were denatured in Lithium dodecyl sulfate (LDS) buffer (NP0007, Thermo Fisher, Darmstadt, Germany) containing 100 mM DTT and separated using NuPAGE Bis-Tris 12% gel (Thermo Fisher, NP0349BOX). Blots were incubated overnight at 4 °C with the following primary antibodies: Anti-pro-brain-derived neurotrophic factor (BDNF) (1:500, Sigma-Aldrich, P1374-200UL), anti-nerve growth factor (NGF) (1:1000 Abcam, ab6199), anti-DARPP-32 (1:5000, Epitomics, 1710-1), anti-tyrosine hydroxylase (TH) at a concentration of 1:1000 (Merck Millipore, AB1542), anti-Iba1 at a concentration of 1:1000 (Wako, 019-1974), and anti-beta actin (1: 5000, Sigma-Aldrich, A5441). Florescence-conjugated secondary antibodies, anti-rabbit and anti-mouse at a dilution of 1:10000 (Li-COR Bioscience, 926-32211 and 926-68070), were used to detect the signals utilizing Li-COR Odyssey imaging system (Li-COR Bioscience).

### 2.10. Statistical Analysis

Experimental results are expressed as means ± SEM, except for the data on MSC phenotype analysis. Survival curves of the animals were analyzed using log rank test. Behavioral data were analyzed by two-way ANOVA with Tukey’s post hoc test. Data from neuropathological analyses were analyzed by two-tailed student’s *t*-tests for comparison between MSCs-treated and PBS-treated R6/2 mice, and between PBS-treated R6/2 mice and PBS-treated WT mice. A non-parametric Mann–Whitney test was performed for non-Gaussian distributions. A *p* value < 0.05 was considered statistically significant.

## 3. Results

### 3.1. Cell Characterization in Vitro

**Figure 1:**
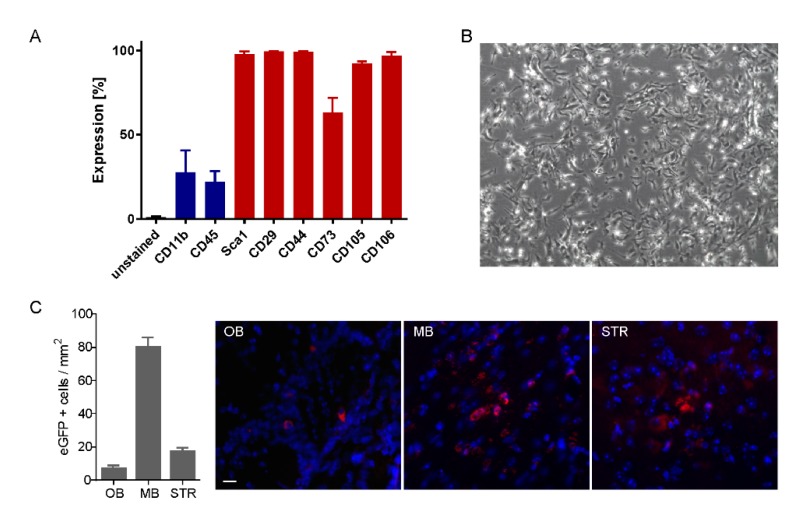
Characterization of mesenchymal stem cells (MSCs) in vitro and MSCs tracking post-delivery. (**A**) MSC phenotype was analyzed before transplantation by means of flow cytometry (*n* = 3, mean ± SD). Blue bars represent negative markers (CD11b and CD45) whereas red bars are the positive markers. (**B**) Exemplary phase contrast image of eGFP-MSC at passage 2. (**C**) Quantification of GFP-positive cells and representative images showing GFP staining (pseudo-colored in red) in the olfactory bulb (OB), midbrain (MB) and striatum (STR) of R6/2 mice 5 days post-delivery of MSCs. Scale bar: 20 µm.

Mouse MSCs were characterized prior to transplantation and found to be positive for the following MSC markers: Sca-1, CD29, CD44, CD73, CD105 and CD106 and negative for hematopoietic markers including CD11b and CD45 (Figure 1A), showing a classical mesenchymal stem cell morphology at passage 4 (Figure 1B). In addition, we confirmed eGFP expression using fluorescence microscopy (Figure S1) and flow cytometry (Figure S2).

### 3.2. Cell Tracking in the Brain after Intranasal Administration

To evaluate the migration of MSCs in different brain regions following intranasal delivery, we investigated the presence of the donor-specific eGFP signal in different brain regions using immunostaining in the mice 5 days (*n* = 3). Five days after the first transplantation, in the entire brain eGFP-expressing MSCs were only found in the midbrain, striatum, and olfactory bulb, whereas the amount of detectable MSCs was much lower in the olfactory bulb compared to the other two brain regions (Figure 1C). The presence of eGFP signal was also investigated 7.5 weeks after MSC administration. No GFP-positive signal was detected in any brain region (data not shown).

### 3.3. Intranasal Administration of MSCs Prolonged Survival of R6/2 Mice with Potentially Improved Motor Function

**Figure 2:**
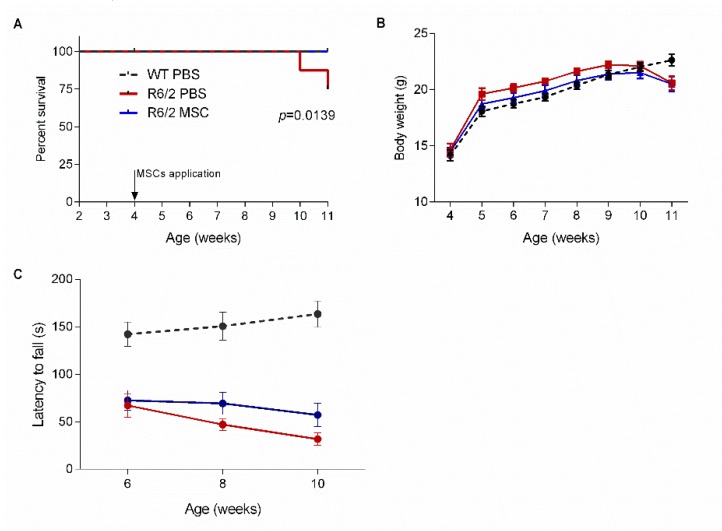
Longitudinal assessment after intranasal MSCs administration. (**A**) Kaplan–Meier survival curve of MSC-treated R6/2 mice and control groups (n = 16 for each group). (**B**) Body weight recorded from 4 to 11 weeks of age (*n* = 16 for R6/2-MSC and wild type (WT)-phosphate-buffered saline (PBS), *n* = 12 for R6/2-PBS). (**C**) Rotarod test performance of MSC-treated mice after MSCs administration (*n* = 16 for R6/2-MSC and WT-PBS, *n* = 14 for R6/2-PBS). R6/2-MSC displayed a trend towards improved latency to fall as compared to R6/2-PBS starting from 4 weeks post MSC delivery (*p* = 0.1059) and continued to 6 weeks after cell application (*p* = 0.0848). Data are expressed as mean ± SEM.

To assess the effect of intranasal administration of MSCs on the survival of R6/2 mice, 16 animals/group were monitored until the end of behavioral tests at 11 weeks of age. The survival curve showed that MSC-treated mice (R6/2-MSC) had a comparable survival rate as WT controls (WT-PBS) (100%), while the R6/2 mice receiving PBS only (R6/2-PBS) exhibited a significantly reduced survival rate of 75% (log rank test, *p* = 0.0139) (Figure 2A). Body weight of mice was monitored weekly. Two-way-ANOVA analysis revealed no significant difference among the 3 treatment groups, although R6/2 mice with PBS or MSCs treatment showed a trend of reduced body weight at 11 weeks of age when compared to WT controls (Figure 2B).

It has been reported that R6/2 mice displayed motor deficits as early as 4 weeks of age as compared to WT littermates [81]. Motor function was assessed by rotarod test at 2, 4 and 6 weeks post intranasal MSCs application. The latency to fall was compared among the 3 treatment groups to evaluate the mice’ performance on the rotating rod. R6/2 mice showed a highly significantly reduced latency to fall during the whole investigation period in comparison to the WT littermates (two-way ANOVA and Tukey‘s post-hoc test, F(1.44) = 27.77, *p* < 0.001). When we only compared the MSC-treated and PBS-treated R6/2 mice using student’s *t*-test, R6/2-MSC displayed a trend towards improved latency to fall as compared to R6/2-PBS starting from 4 weeks post MSC delivery (*p* = 0.1059) and continued to 6 weeks after cell application (*p* = 0.0848). These results suggested a potentially improved motor function in R6/2 mice after intranasal applications of MSCs (Figure 2C).

### 3.4. Ameliorated Circadian Rhythm in the MSC-Treated R6/2 Mice

**Figure 3:**
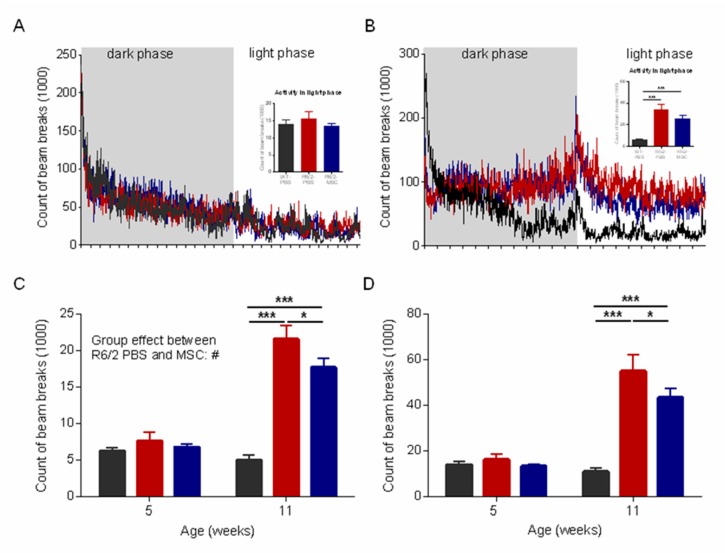
Ameliorated sleeping disturbance in the MSC-treated R6/2 mice at the later disease stage. Locomotor activities of mice were monitored using LabMaster at 5 and 11 weeks of age for 22 h (*n* = 16 for R6/2-MSC and WT-PBS groups, *n* = 12 for R6/2-PBS group). The counts of beam breaks represent the ambulatory activities during the whole recording period (22 h) at (**A**) 5 and (**B**) 11 weeks of age, and (**C**) fine movement and (**D**) total activities in the light phase at both 5 and 11 weeks of age. Data are represented as mean ± SEM. *: *p* < 0.05; ***: *p* < 0.001.

Numerous studies have shown disrupted circadian rhythm in HD patients and animal models including R6/2 mice [82,83,84]. We tracked the locomotor behavior of the animals for 22 h (12 h dark phase and 10 h light phase) using LabMaster to evaluate their activities and circadian rhythms at 1 and 7 weeks after cell administration (i.e., 5 and 11 weeks of age, respectively). At 11 weeks of age, R6/2 mice with either MSC or PBS treatment showed an abnormal circadian rhythm with increased ambulatory activity during the light phase as compared to WT controls, although this phenotype was not observed at 5 weeks of age (1 week after cell administration) (Figure 3A,B). We therefore compared the sum of fine movement and total activity over the light phase. Two-way ANOVA and Tukey‘s post-hoc test revealed that both were significantly reduced in the MSC-treated R6/2 mice compared to R6/2-PBS mice at 11 weeks of age (*p* < 0.05 for both) (Figure 3C,D).

### 3.5. Gene Expression Profiles of Inflammatory Regulators and Neurotrophic Factors

**Figure 4:**
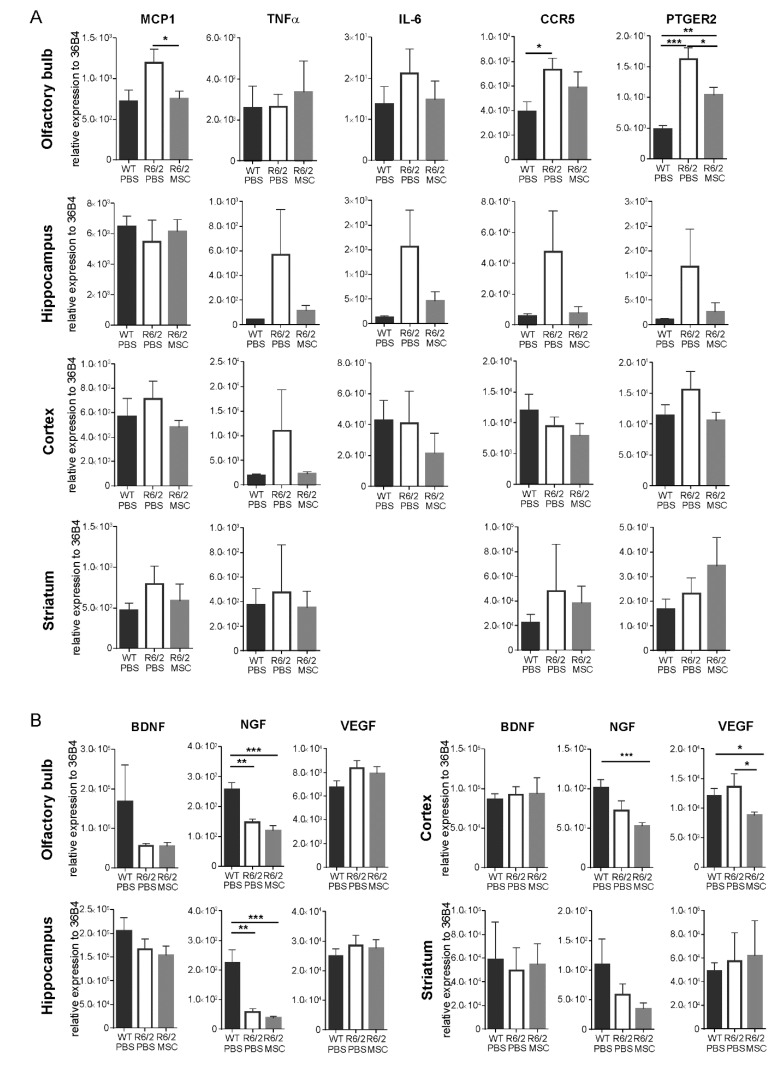
Altered gene expression of inflammation markers and neurotrophic factors in different brain regions. mRNA expression levels of (**A**) inflammatory regulators (MCP1, TNFα, IL-6, CCR5, and PTGER2) and (**B**) neurotrophic factors (BDNF, VEGF, and BDNF) were analyzed in 4 different brain parts (olfactory bulb, hippocampus, cortex and striatum) (WT-PBS, *n* = 6; R6/2-PBS, *n* = 6 and R6/2-MSC *n* = 8). Values were normalized to 36B4 level. Data are presented as mean ± SEM. IL-6 was not detectable in the striatum and hence was not presented here. *: *p* < 0.05; **: *p* < 0.01; ***: *p* < 0.001.

We analyzed the gene expression levels of inflammatory regulators and neurotrophic factors in the olfactory bulb, hippocampus, striatum and cortex at 11.5 weeks of age (7.5 weeks post-application of MSCs) (R6/2-MSC, *n* = 8, R6/2-PBS, *n* = 6 and WT-PBS, *n* = 6). Analyses of the gene expression levels of the inflammatory regulators including macrophage chemoattractant protein (MCP1), TNFα, interleukin-6 (IL-6), C-C chemokine receptor type 5 (CCR5) and prostaglandin E2 receptor (PTGER2) revealed that these genes showed a general trend of increase in expression in the R6/2-PBS mice with the exception of MCP1 in hippocampus and IL-6 and CCR5 in cortex as compared to WT-PBS mice, and these aberrant increase in gene expressions were restored in the R6/2-MSC mice to comparable levels of the WT-PBS mice (Figure 4A). In particular, when compared to the WT-PBS group, CCR5 (student’s *t*-test, *p* < 0.05) and PTGER2 (student’s *t*-test, *p* < 0.01) were significantly upregulated in the olfactory bulb of R6/2-PBS, while MSC treatment in R6/2 mice (R6/2-MSC) led to a significant downregulation of MCP1 (student’s *t*-test, *p* < 0.05) and PTGER2 (student’s *t*-test, *p* < 0.05) gene expressions in the same brain area. However, such differences were neither detected in the striatum nor the cortex.

We also analyzed the gene expression levels of the neurotrophic factors, such as brain derived neurotrophic factor (BDNF), nerve growth factor (NGF) and vascular endothelial growth factor (VEGF). In comparison with WT-PBS, NGF was downregulated in all investigated brain regions of R6/2-PBS mice although the decrease did not reach statistical significance in cortex and striatum. MSC treatment (R6/2-MSC) further suppressed the mRNA expression of NGF in olfactory bulb, hippocampus and cortex. On the other hand, the expression of BDNF and VEGF were not significantly different among the 3 treatment groups in all analyzed brain regions although BDNF protein has been reported to be reduced in HD mouse brains [85] (Figure 4B). We have hence quantified the protein expression of BDNF in the hippocampus and cortex 7.5 weeks post-transplantation. Our results demonstrate that neither the glycosylated nor the non-glycosylated form of BDNF showed a significant difference among the treatment groups in the hippocampus (Figure S3A). In the cortex, the non-glycosylated form of BDNF was reduced in the R6/2-PBS mice when compared to the WT-PBS group (student’s *t*-test, *p* < 0.01), whereas no change was found between MSC-treated and non-treated R6/2 mice (Figure S3B).

### 3.6. Microglial Changes in MSC-Treated R6/2 Mice

**Figure 5:**
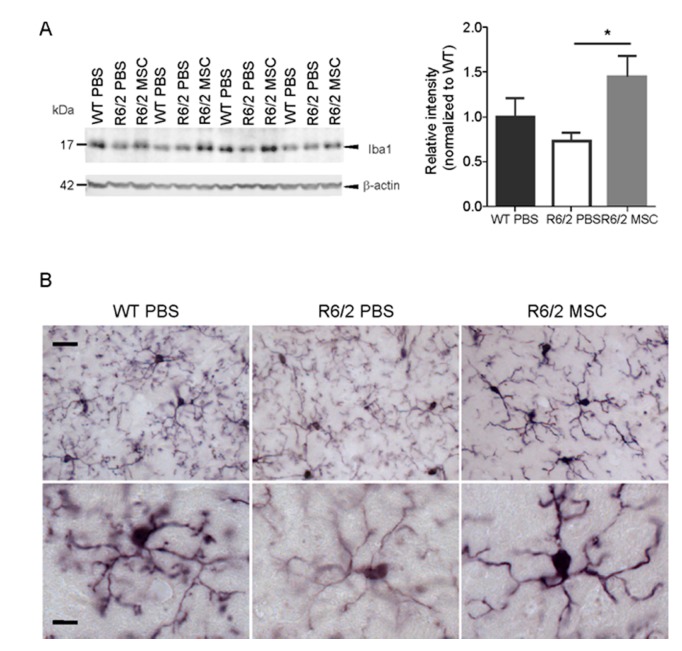
Enhanced expression of Iba1 and morphological changes of striatum-resident microglia in MSC-treated R6/2 mice. (**A**) Quantification of Iba1 protein expression level in the striatum using western blot. Intensity of Iba1-specific band at 17 kDa was compared among R6/2-MSC, R6/2-PBS and WT-PBS (*n* = 4 for each group) 7.5 weeks after MSC application. Values were normalized to the level of ß-actin in each lane. Statistical analysis was performed using the student *t*-test. Data are presented as mean ± SEM *: *p* < 0.05. Full western blots are shown in Figure S5A. (**B**) Representative images of Iba1 staining of striatum-resident microglia in the brain samples obtained in parallel to those analyzed using the western blot. When compared to WT-PBS mice, microglia of R6/2-PBS mice had thinner processes, less process ramification and reduced Iba1 immunoreactivity, whereas MSC treatment (i.e., R6/2-MSC mice) restored Iba1 expression and the thickness of microglial processes. Scale bar in the upper panel: 20 µm, in the lower panel: 8 µm.

Analyses of the protein expression level of the microglial marker Iba1 in the striatum using western blot (11.5 weeks of age, *n* = 4 for each group) revealed an increased Iba1 in the R6/2-MSC mice compared to R6/2-PBS mice (student *t*-test, *p* < 0.05), while no significant difference was found between WT-PBS and R6/2-PBS control groups (Figure 5A). Morphological changes of microglia were examined using immunohistological staining with antibody against Iba1. In agreement with a previous report [86], microglial structural abnormalities such as thinner processes, decreased ramification and reduced Iba1 immunoreactivity were observed in R6/2-PBS mice compared to the WT-PBS littermates at 11.5 weeks. In contrast to R6/2-PBS mice, microglia of R6/2-MSC mice displayed increased process thickness and enhanced Iba1 immunoreactivity (Figure 5B).

### 3.7. Neuropathological Changes in MSC-Treated R6/2 Mice

**Figure 6:**
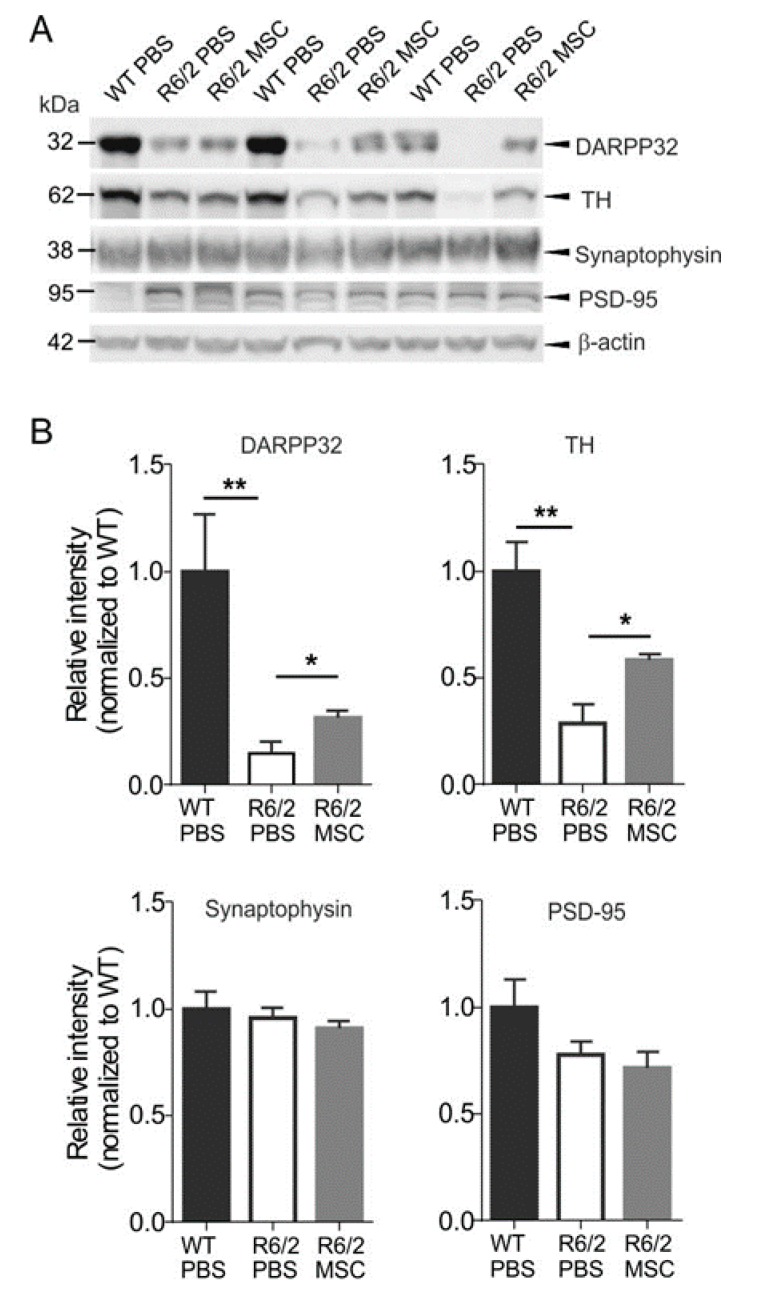
(**A**) Increased expression levels of DARPP-32 and tyrosine hydroxylase (TH) in the striatum of MSC-treated mice. The protein levels of DARPP-32 and TH were analyzed using mice striatal lysates and compared among R6/2-MSC, R6/2-PBS and WT-PBS (*n* = 4 for each group) 7.5 weeks after MSC application. (**B**) Both DARPP-32 and TH showed significantly reduced levels in R6/2-PBS mice as compared to WT-PBS mice (student’s *t*-test), whereas these reductions were ameliorated as R6/2-MSC mice exhibited higher expression levels of DARPP-32 and TH (student’s *t*-test). There is no difference in protein expression level of synaptic markers synaptophysin and PSD-95 among all three groups. Data are represented as mean ± SEM. *: *p* < 0.05; **: *p* < 0.01. Full western blots are shown in Figure S5B.

As the striatum is the most affected brain region in HD, it is crucial to investigate the effects of intranasal MSC administration on neuronal survival in the striatum. DARPP-32, a widely used marker of mature medium spiny neurons (MSNs), has been reported to be reduced in the striatum of R6/2 mice as compared to WT littermates, indicating neuronal loss and dysfunction of MSNs in the striatum [87,88,89]. Hence, we quantified the protein levels of DARPP-32 in the WT-PBS, R6/2-PBS and R6/2-MSC groups using western blotting 7.5 weeks after MSC administration (11.5 weeks of age, *n* = 4 for each group) (Figure 6A). In agreement with previous studies [87,88,89], R6/2-PBS mice showed a strongly reduced protein level of DARPP-32 as compared to WT-PBS controls (student’s *t*-test, *p* < 0.01), while R6/2-MSC mice exhibited a significantly increased DARPP-32 level when compared to R6/2-PBS mice (student’s *t*-test, *p* < 0.05) (Figure 5A). This result was verified by immunofluorescence staining as indicated in the representative images of immunoreactivity of DARPP-32 in the striatum (Figure 6B). We have also investigated the protein expression levels of TH, the rate-limiting enzyme for dopamine biosynthesis, in the striatum of the same cohort. Consistent with a previous report [90], the expression level of TH in the striatum of R6/2-PBS mice was significantly reduced as compared to WT-PBS mice (student’s *t*-test, *p* < 0,01), and this reduction was significantly attenuated in the MSC-treated group (student’st *t*-test, *p* < 0.05) (Figure 6A). We further quantified the protein expression levels of the synapse markers synaptophysin and PSD-95, and no significant difference could be detected among the treatment groups (Figure 6). Altogether, these results demonstrated an amelioration of the changes in the dopaminergic pathway in MSC-treated R6/2 mice via intranasal delivery.

As previous studies on intrastriatal administration of MSCs in HD animal models have reported the beneficial effect of MSCs might be associated with a decrease in mHTT aggregates formation [91,92], we analyzed mHTT aggregation using immunohistological staining with EM48 at the age of 11.5 weeks (*n* = 4 for each group). While R6/2-PBS mice displayed abundant nuclear inclusion bodies and neuropil aggregates in the striatum, we could not detect any difference in the abundance of nuclear inclusion bodies and neuropil aggregates in the striatum of the MSC-treated mice (Figure S4). Brain volume was also quantified using the same cohort by stereology. Mean striatal area of 6 consecutive brain sections with a 150 µm interval (starting from Bregma 0.98) was analyzed and revealed no difference between R6/2-MSC and R6/2-PBS mice (data not shown).

## 4. Discussion

The main findings of the present study are: (1) MSCs delivered intranasally to R6/2 HD mice were able to migrate to and infiltrate into the olfactory bulb, midbrain and striatum 5 days post-delivery; (2) intranasal delivery of MSCs significantly increased survival rate and ameliorated sleep disturbance of R6/2 mice as well as showing a trend towards improved motor function; (3) MSCs treatment in R6/2 mice increased DARPP-32 expression in the striatum while the expression levels of synaptic markers and NeuN remained unchanged; (4) all investigated immunomodulators were either significantly restored or showed a trend towards restoration in most of the brain areas examined after MSCs treatment; and (5) neuroprotective effects of MSC were concomitant with increased expression of Iba1 in the striatum and restored morphology of striatum-resident microglia of R6/2 mice.

### 4.1. Migration Pattern and Survival of Intranasally Delivered MSCs in the Brain

Our results of cell tracking 5 days after intranasal delivery showed that the applied MSCs were distributed among the olfactory bulb, midbrain and striatum. This indicates that exogenous MSCs were able to migrate to the brain shortly after being delivered from the nose along the olfactory and trigeminal nerve pathways in R6/2 mice as reported previously for intranasal delivery of stem cells [41,44,92,93,94] and drugs or biologics in different models of CNS disorders [95,96,97]. MSCs were exclusively found in the striatum, olfactory bulb and midbrain 5 days post-transplantation, and they were more abundant in the midbrain than in the olfactory bulb. This more caudally directed distribution of MSC suggests their preferential migration to the lesioned regions as previously shown by intravenous administration of MSC in a model of brain injury [98]. Another explanation for rapid appearance of MSCs in deeper parts of the brain, such as striatum and midbrain, is their transportation via CSF, once they entered the subarachnoid space after crossing the cribriform plate as described previously [41]. It cannot be excluded that a portion of cells could reach the CNS via blood stream by entering the blood vessels of the nasal mucosa. However, in line with our observations, none of the previous studies could show intranasally delivered stem cells within the lumen of cerebral vessels [41,43,44,92,93,94].

Investigation on the engrafted MSCs 7.5 weeks post-cell-administration showed no detectable GFP signal in any brain area indicating a poor long-term survival rate as reported in previous studies [40,99]. In contrast, we found a wide range of readouts that were ameliorated including neuropathological and neurobehavioral changes at/until this time point. Although MSC possess the capacity of transdifferentiation to various cell types, a therapeutic effect has been proposed to be contributed by the secretion of vesicles and other molecules including cytokines and chemokines (reviewed in [100]). This hypothesis is supported by numerous pre-clinical studies demonstrating therapeutic effect upon administration of MSC-conditioned medium or -produced exosome [101,102,103]. Particularly, a study using a rat model with overactive bladder demonstrated increases of primitive progenitor cells genes and genes involved in stem cell trafficking processes in the bladder tissue transplanted with MSCs but no engraftment [104]. This finding suggests the activation of primitive progenitor cells by MSC paracrine effect as a possible mechanism for long-term therapeutic efficacy of MSCs.

### 4.2. Increased TH and DARPP-32 Expressions and Attenuated Circadian Rhythm Disturbances Indicate An Amelioration of Dopamine Signaling in MSC-Treated Mice

In this study, MSC treatment resulted in increased TH and DARPP-32 protein expressions, both of which are involved in dopamine biosynthesis and neurotransmission. As in HD patients, R6/2 mice displayed a decreased TH expression as its transcription was disrupted by mutant huntingtin [90]. Similarly, the immunoreactivity of DARPP-32 in the striatum had been reported to be reduced by approximately 50% even in the presymptomatic R6/2 mice as compared to WT animals [75,105] although the number of neurons in the striatum remained unaltered. In the dopaminergic pathway, TH is the rate-limiting enzyme for the conversion of tyrosine into the precursor of dopamine (i.e., L-3,4-dihydroxyphenylalanine (L-DOPA)), whereas DARPP-32 phosphorylation is bi-directionally modulated by dopamine receptors 1 and 2 in the neostriatum [106]. As a result, the reduction of TH and DARPP-32 expressions led to the impairment of dopaminergic signaling cascade [75]. This was rescued, at least partially, by MSC treatment, as demonstrated by the tendentially improved motor ability of the MSC-treated R6/2 mice. Another important behavioral improvement observed in MSC-treated R6/2 mice was their circadian activity pattern. Consistent with previous studies [57,83], our analyses showed that R6/2 mice suffered from sleep disturbance as they exhibited aberrant patterns of fine movement and ambulatory activities in light-dark phases, whereas MSC treatment markedly alleviated the disruption of the sleep-wake cycle. In mammals, the circadian clock is centrally regulated in the suprachiasmatic nuclei (SCN) [107,108,109] with an array of circadian genes widely expressed across the whole brain. Among these genes, the expressions of Per1 [110] and Per2 [83,111] are mediated by dopamine signaling. In particular, mPer2 expression was found to be significantly altered in the forebrain [57] and SCN [83] in R6/2 mice. As we have shown that the dopaminergic system in R6/2 mice benefited from the MSCs treatment, although we did not pursue deeper into the precise molecular mechanisms of MSCs treatment on circadian control in this study, the remedial effects of MSCs suggest a causal link between MSCs and circadian rhythm correction, probably via the restoration of functional dopamine signaling on circadian genes induction/expression. Another possible explanation could be the regulation of circadian genes by inflammatory cytokines [112,113], for instance, IL-6 is known to suppress the circadian clock [114].

### 4.3. Intranasal Administration of MSCs Reduced Neuroinflammation

As in HD patients [115], inflammatory factors are up-regulated in R6/2 mice [59]. In line with these studies, our data also showed trends of increased transcription of inflammatory modulators (MCP1, CCR5, IL6, PTGER2 and TNFα) in different brain regions of R6/2 mice. Intranasal administration of MSCs in R6/2 mice suppressed most of these abnormally up-regulated gene expressions attributed to the immunomodulatory properties of MSCs [68,116,117,118,119], and such immunomodulatory capacity was further enhanced in the inflammatory conditions [118,120,121]. Substantiated by the restored expressions of the investigated inflammatory modulators, our study validated the immunoregulatory ability of MSCs in HD as in other disorders [44,80,94]. Another neuroprotective potential of MSC is the secretion of neurotrophic factors, which has been reported in numerous studies including several MSC therapies for HD [91,121,122,123]. However, we did not detect any increased expressions of neurotrophic factors in R6/2-MSC mice as compared to the R6/2-PBS control group.

Interestingly, our results revealed an increased protein expression level of microglia marker Iba1 in MSC-treated R6/2 mice, indicating an activation of microglia, in contrast to the results of the ameliorated inflammatory modulators. Although it is a common feature that Iba1 expression is increased in both HD patients and symptomatic HD animal models, its expression is decreased in the pre-symptomatic stage of R6/2 mice [74]. Moreover, impaired migration and function of microglia have been reported in YAC128 and BACHD mice in response to brain injury [73]. These evidences suggest that mutant huntingtin protein affects microglial function under both basal and inflammatory conditions. Other reports showed that supplementation of normal microglia increased survival rate and electrophysiological properties of neurons expressing mHTT in vitro [124] and in vivo [125]. Since MSCs modulate the functional properties of microglia via TGF-β [126], TSG-6 [127], CX3CL1 [128], all of which are pro-inflammatory molecules, and microvesicles [129], MSCs could lead to microglia activation as shown by the increase in Iba1 expression in the MSC-treated R6/2 mice. In addition, it has been shown that the introduction of MSCs to primary rat microglia led to a shift of the active microglia phenotype from classical M1 to alternative M2 in vivo [126]. M1 secretes proinflammatory cytokines causing toxic effects, whereas M2 promotes neuronal protection by releasing neurotrophic factors that led to reduced proinflammatory cytokines [130]. Besides, microglia are also involved in the modulation of synaptic plasticity and transmission (reviewed in [131]), its alteration potentially also contributes to the ameliorated dopamine transmission. In addition, our data demonstrate a thinning of microglial processes in the R6/2 mouse model of HD similar to that of transgenic Alzheimer’s disease mice, which has been suggested to be associated with impaired microglial function [132]. This microglial morphology alteration has been successfully ameliorated by intranasal MSC treatment in R6/2 mice.

It is interesting to compare the treatment outcome of MSCs administrated via intranasal administration (INA) as an alternative non-invasive delivery route with MSCs applied via stereotactic injection, which directly delivers cells to the most affected brain regions. Intrastriatal injection of bone marrow-derived MSCs at low passage (3–8) in R6/2 mice had a short two-week effect on spatial memory, while injection of MSCs at high passage (40–50) had a significant additional effect on rotarod performance and neuronal metabolism [123]. Another study reported an improved performance on the rotarod and increased striatal numbers of neurons in YAC128 HD mice injected with genetically engineered bone-marrow-derived MSCs that over-express BDNF, but these therapeutic effects were not observed in those injected with normal MSCs [133]. In comparison, the present study demonstrated the amelioration of both the behavioral phenotype and neuropathological changes in R6/2 HD mice after administration of bone-marrow-derived MSCs via INA. Moreover, MSCs were found in several major brain regions such as the olfactory bulb and striatum, suggesting a beneficial treatment effect attributed to multiple brain areas in intranasally treated mice.

## 5. Conclusions

Our results demonstrate significantly ameliorated behavioral and neuropathological phenotypes of R6/2 mice after intranasal MSC administration. This indicates that this method is an effective route for delivering MSCs for CNS-targeted HD therapy. Being non-invasive, intranasal delivery of MSCs can be repeatedly applied, resulting in a long-lasting therapeutic effect, overcoming the challenge of low cell survival and host immune response after surgical administration.

## Supplementary Materials

The following are available online at https://www.mdpi.com/2073-4409/8/6/595/s1. Figure S1: Exemplary fluorescence images of cultured MSCs at passage 1, Figure S2: Exemplary flow cytometry data, Figure S3: Western blot analyses of protein expression levels of glycosylated and non-glycosylated BDNF in the hippocampus and cortex of mice at 11.5 weeks of age, Figure S4: Representative images of the staining of mutant huntingtin aggregates in the striatum of MSC-treated R6/2 mice and control groups, Figure S5: Full western blots used in Figure 5 and Figure 6.
