# Supplementary material for: Intranasal Administration of Mesenchymal Stem Cells Ameliorates the Abnormal Dopamine Transmission System and Inflammatory Reaction in the R6/2 Mouse Model of Huntington Disease
Source: Cells. 2019 Jun 15;8(6):595. doi: 10.3390/cells8060595 (PMC6628278; doi:10.3390/cells8060595)
Supplement: Supplementary file 1 [file cells-08-00595-s001.pdf]

cells

MDPI

Supplementary material

Intranasal administration of mesenchymal stem cells ameliorates the abnormal dopamine transmission system and inflammatory reaction in the R6/2 mouse model of Huntington disease

Libo Yu-Taeger[1,2#], Janice Stricker-Shaver[1,2#], Katrin Arnold[3,4], Patrycja Bambynek-Dziuk[1,2], Arianna Novati[1,2], Elisabeth Singer[1,2], Ali Lourhmati[5], Claire Fabian[3,4], Janine Magg[1,2], Olaf Riess[1,2], Matthias Schwab[5,6,7,8], Alexandra Stolzing[3,9], Lusine Danielyan[5,7,8] and Hoa Huu Phuc Nguyen[1,2,7,10,11*]

# contributed equally

[1] Institute of Medical Genetics and Applied Genomics, University of Tuebingen, D-720176 Tuebingen, Germany; Libo.Yu-Taeger@med.uni-tuebingen.de (L.YT.); Janice.Stricker-Shaver@med.uni-tuebingen.de (J.SS.); Elisabeth.Singer@med.uni-tuebingen.de (E.S.); Patrycja.Bambynek-Dziuk@med.uni-tuebingen.de (P.BD.); Arianna.Novati@med.uni-tuebingen.de (A.N.); Janine.Magg@med.uni-tuebingen.de (J.M.); Olaf.Riess@med.uni-tuebingen.de (O.R.);
[2] Centre for Rare Diseases (ZSE), University of Tuebingen, D-72076 Tuebingen, Germany
[3] Interdisciplinary Centre for Bioinformatics (IZBI), University of Leipzig, D-04107 Leipzig, Germany; katrin.arnold@izi.fraunhofer.de (K.A.);
[4] Fraunhofer Institute for Cell Therapy and Immunology (IZI), D-04103 Leipzig, Germany; claire.fabian@izi.fraunhofer.de (C.F.);
[5] Department of Clinical Pharmacology, University Hospital of Tuebingen, D-72076 Tuebingen, Germany; alilourhmati@yahoo.de (A. L.); Lusine.Danielyan@med.uni-tuebingen.de (L.D.);
[6] Dr. Margarete Fischer-Bosch Institute of Clinical Pharmacology, D-70376 Stuttgart, Germany; Matthias.Schwab@ikp-stuttgart.de (M.S.);
[7] Departments of Biochemistry and Clinical Pharmacology, Yerevan State Medical University, 0025 Yerevan, Armenia;
[8] Laboratory of Neuroscience, Yerevan State Medical University, 0025 Yerevan, Armenia;
[9] Centre for Biological Engineering, School of Mechanical, Electrical and Manufacturing Engineering, Loughborough University, Loughborough LE11 3TU, UK; A.Stolzing@lboro.ac.uk (A.S.);
[10] Department of Human Genetics, Ruhr University of Bochum, D-44801 Bochum, Germany; huu.nguyen-r7w@rub.de (H.H.P.N.);
[11] Departments of Medical Chemistry and Biochemistry, Yerevan State Medical University, 0025 Yerevan, Armenia.

* Correspondence: huu.nguyen-r7w@rub.de; Tel.: +49 234 32 23839

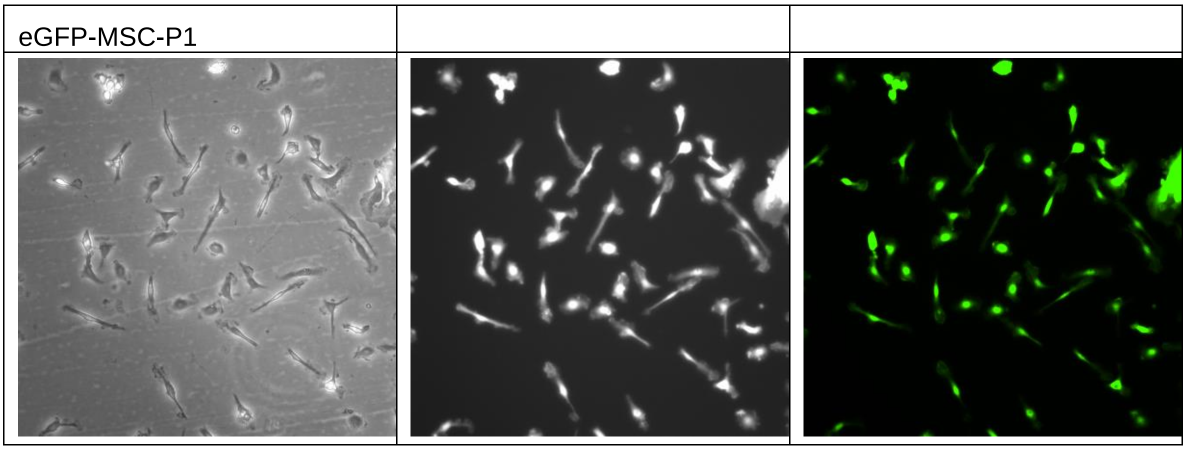

Figure S1.

Exemplary fluorescence images of cultured MSCs at passage 1.

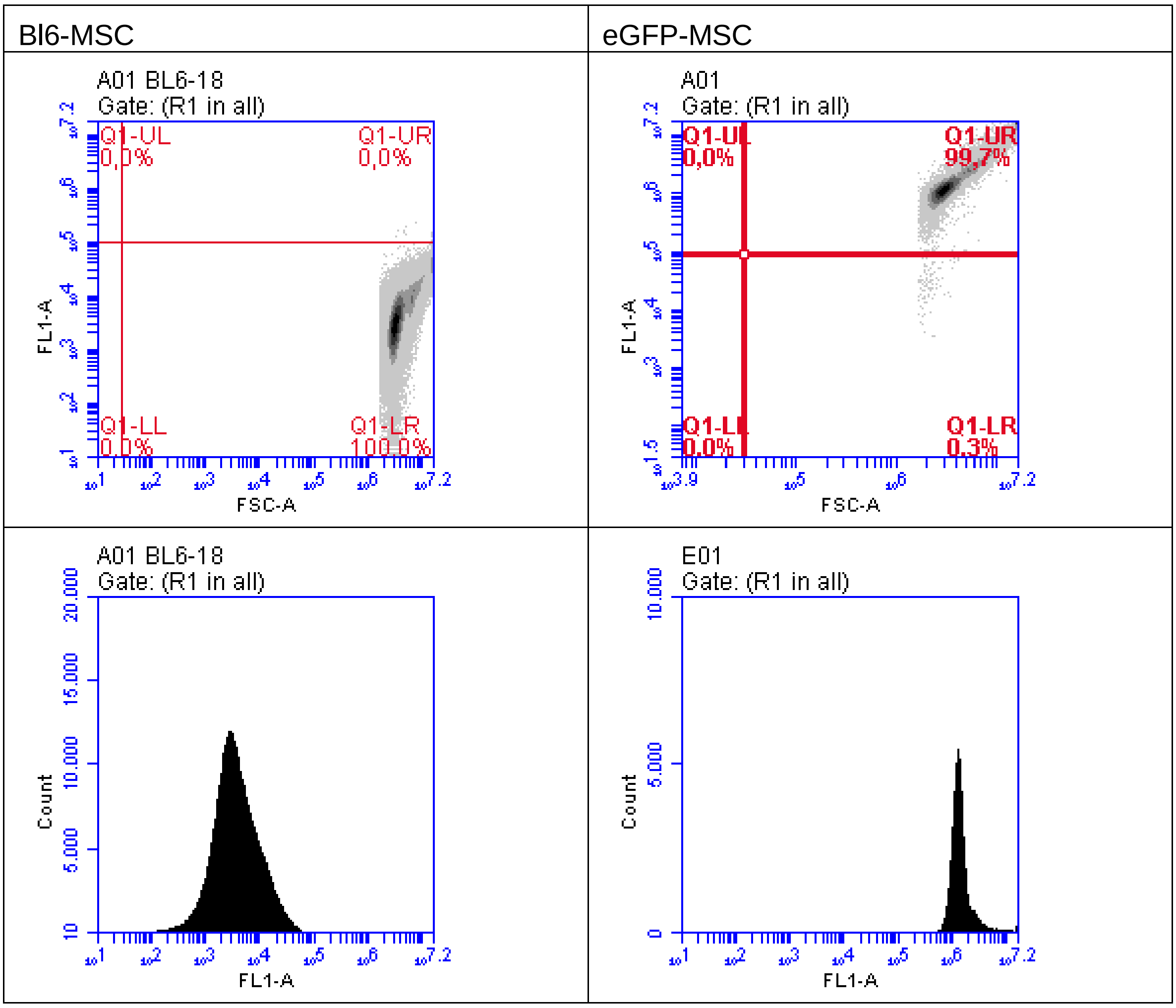

Figure S2.

Exemplary flow cytometry data: eGFP-MSCs show a much higher Fl1-signal compared to Bl6-MSCs, shown in Density plots and histogram.

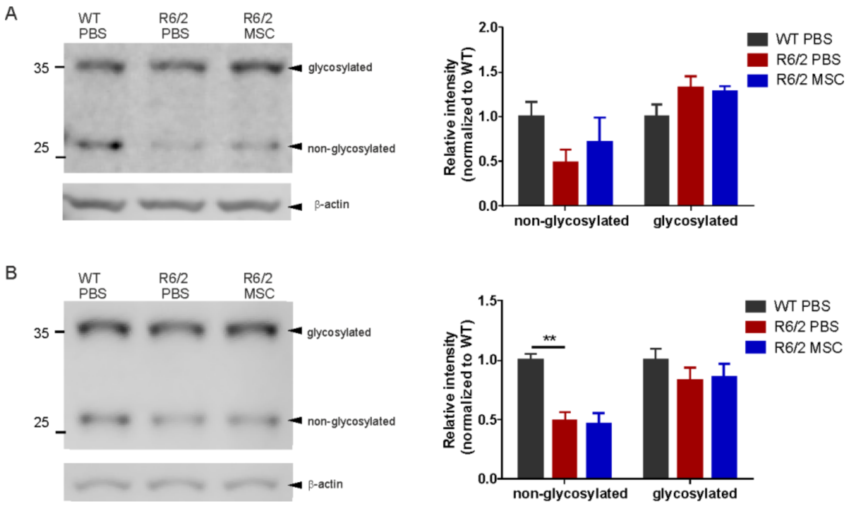

Figure S3.

Western blot analyses of protein expression levels of glycosylated and non-glycosylated BDNF in the hippocampus and cortex of mice at 11.5 weeks of age (7.5 weeks post-MSCs delivery) (n=4). **(A)** Quantification of density of bands representing glycosylated and non-glycosylated BDNF showed no significant difference among the treatment groups in the hippocampus (one-way ANOVA, p>0.05). (B) In the cortex, the non-glycosylated form of BDNF was reduced in the R6/2-PBS mice when compared to the WT-PBS group (Student *t*-test, p<0.01), whereas no change was found between MSC-treated and non-treated R6/2 mice. Data are presented as mean ± SEM.

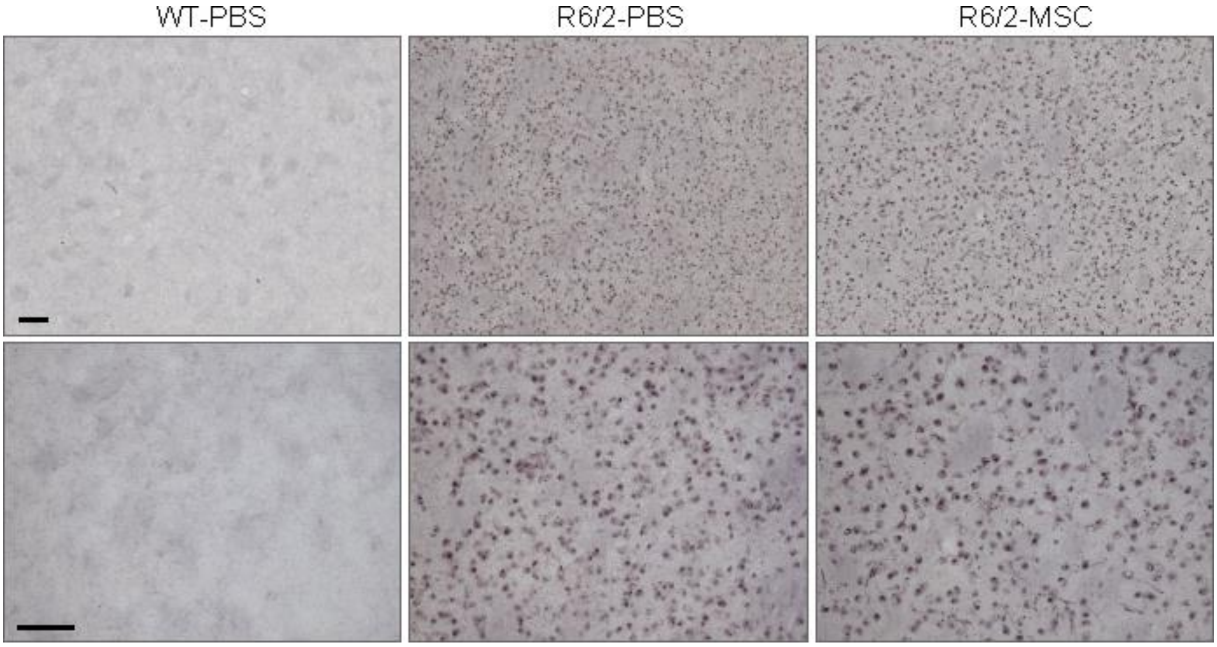

Figure S4.

Representative images of the staining of mutant huntingtin aggregates in the striatum of MSC-treated R6/2 mice and control groups (at the magnification of 20X (lower panel) and 40X (upper panel)). Coronal cryosections of brains from mice at 11.5 weeks of age (7.5 weeks post MSCs delivery) were stained with monoclonal antibody EM48, which recognizes the N-terminal region of human huntingtin (n=4). Abundance of huntingtin aggregates in MSC-treated R6/2 and R6/2-PBS groups was comparable, while WT mice did not show any EM48 immunoreactivity. Scale bars: 100 µm

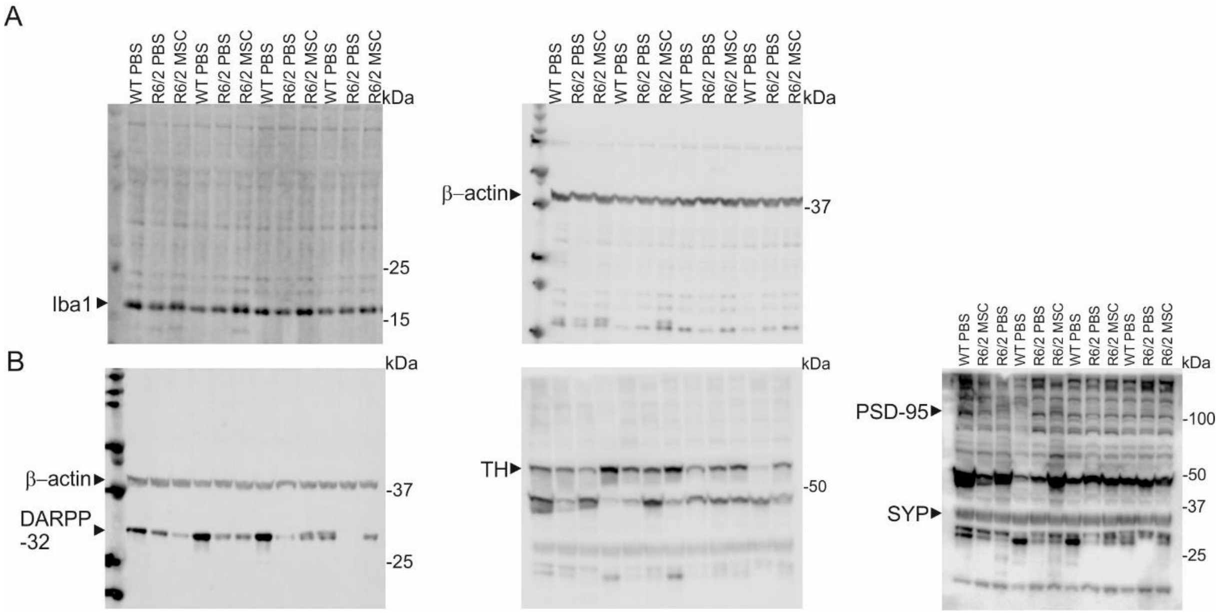

Figure S5.

Full western blots used in Figure 5 and Figure 6. Not all individual steps of signal acquisition are shown in (B). (A) Ionized calcium binding adapter molecule 1 (Iba1) and β-actin as loading control. (B) Dopamine and cyclic AMP-regulated phosphoprotein (DARPP-32), β-actin as loading control; tyrosine hydroxylase (TH); postsynaptic density protein 95 (PSD-95) and synaptophysin (SYP).
